# Kleptoplasty does not promote major shifts in the lipidome of macroalgal chloroplasts sequestered by the sacoglossan sea slug *Elysia viridis*

**DOI:** 10.1038/s41598-017-12008-z

**Published:** 2017-09-13

**Authors:** Felisa Rey, Elisabete da Costa, Ana M. Campos, Paulo Cartaxana, Elisabete Maciel, Pedro Domingues, M. Rosário M. Domingues, Ricardo Calado, Sónia Cruz

**Affiliations:** 1 Departamento de Biologia & CESAM & ECOMARE, Universidade de Aveiro, Campus Universitário de Santiago, 3810-193 Aveiro, Portugal; 2 Centro de Espetrometria de Massa, Departamento de Química & QOPNA, Universidade de Aveiro, Campus Universitário de Santiago, 3810-193 Aveiro, Portugal

## Abstract

Sacoglossan sea slugs, also known as crawling leaves due to their photosynthetic activity, are highly selective feeders that incorporate chloroplasts from specific macroalgae. These “stolen” plastids - kleptoplasts - are kept functional inside animal cells and likely provide an alternative source of energy to their host. The mechanisms supporting the retention and functionality of kleptoplasts remain unknown. A lipidomic mass spectrometry-based analysis was performed to study kleptoplasty of the sacoglossan sea slug *Elysia viridis* fed with *Codium tomentosum*. Total lipid extract of both organisms was fractionated. The fraction rich in glycolipids, exclusive lipids from chloroplasts, and the fraction rich in betaine lipids, characteristic of algae, were analysed using hydrophilic interaction liquid chromatography-mass spectrometry (HILIC-LC-MS). This approach allowed the identification of 81 molecular species, namely galactolipids (8 in both organisms), sulfolipids (17 in *C. tomentosum* and 13 in *E. viridis*) and betaine lipids (51 in *C. tomentosum* and 41 in *E. viridis*). These lipid classes presented similar lipidomic profiles in *C. tomentosum* and *E. viridis*, indicating that the necessary mechanisms to perform photosynthesis are preserved during the process of endosymbiosis. The present study shows that there are no major shifts in the lipidome of *C. tomentosum* chloroplasts sequestered by *E. viridis*.

## Introduction

Sacoglossan sea slugs have been popularly termed as crawling leaves^1^ due to their singular relationship with their food. This group of sea slugs are highly specialized feeders, using their radular teeth to penetrate the cell wall of siphonaceous algae and suck the entire cytosolic content^2^. While the whole cellular content of these algae, including the nucleus, is digested, chloroplasts are sequestered by the sea slugs. These “stolen” organelles, also known as kleptoplasts^3^, pass through the sea slugs’ gut and are phagocytized into the digestive epithelium. Inside animal cells, kleptoplasts are in direct contact with the cytosol, being able to keep their structural integrity and functionality for variable periods (from hours to months)^4^. Therefore, these sea slugs are mixotrophic animals, with their energy being secured through heterotrophic and autotrophic pathways^1, 5^.

The mechanisms supporting the retention and functionality of chloroplasts inside sea slug cells are still unknown. Nevertheless, a recent study revealed that the abundance of kleptoplasts in juveniles of *Elysia chlorotica* is related with the abundance of lipid droplets^6^. This study proposes a protective mechanism of stolen plastids by the lipids generated by the kleptoplasts, which involves stabilization of plastids and their long-term retention^6^. The relationship between plastids and lipids is not totally new, as Trench *et al*.^7^ already identified lipid trafficking from functional plastids to the sea slug *E. viridis*. Lipids are important components in all organisms, as source of metabolic energy and essential constituents of biological membranes. Although little attention has been given to the role of lipids in kleptoplasty, several studies have recognized the relevant role of these molecules in the process of endosymbiosis in other marine biological interactions^8–10^. These studies have identified significant changes in the host lipidome during the establishment of symbionts^8, 11^, suggesting an active role of lipids in this process.

**Figure 1:**
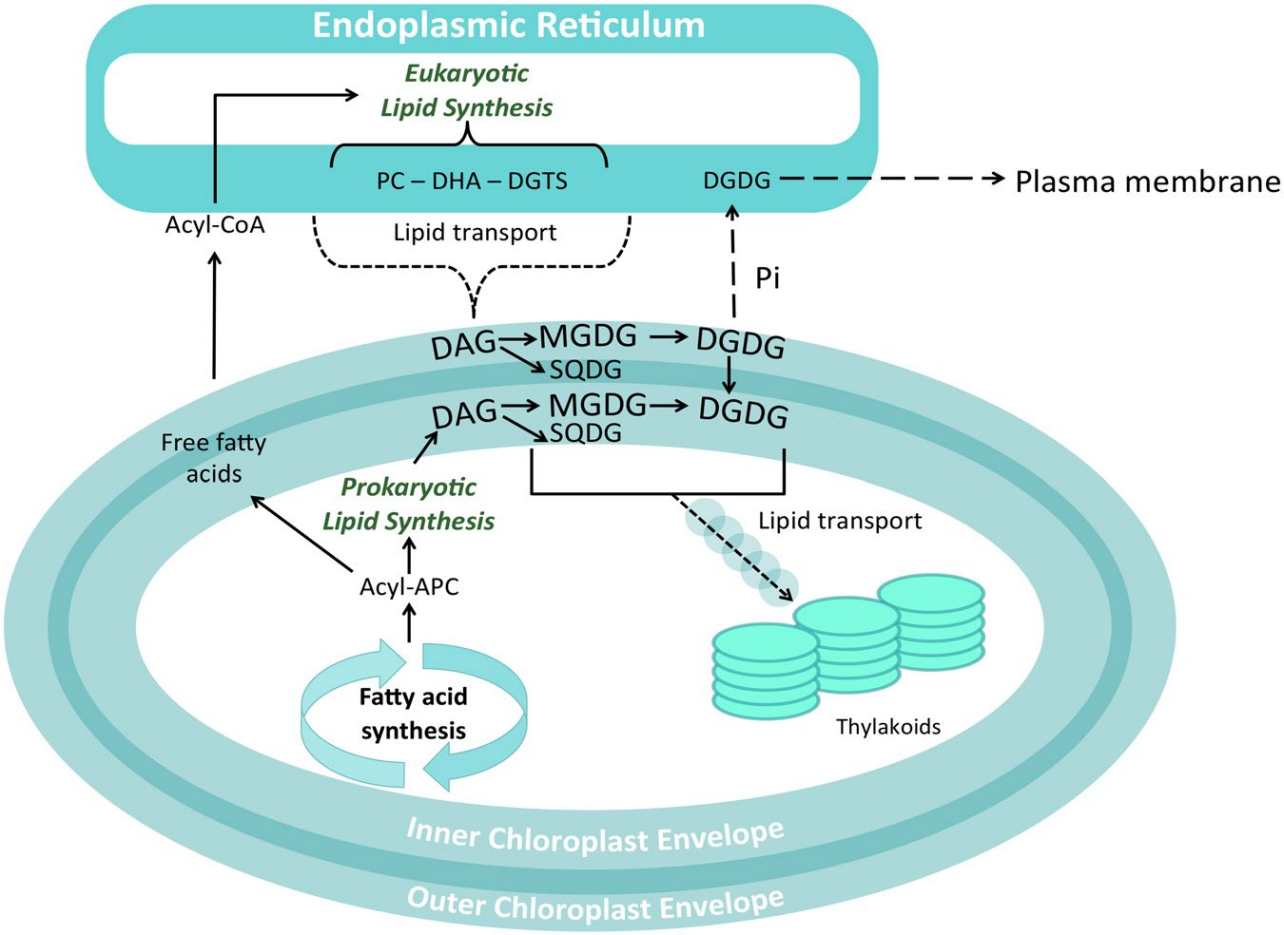
Schematic representation of lipid synthesis in chloroplasts. Lipids can be synthesized entirely within the chloroplast - prokaryotic lipid synthesis - or in collaboration with the endoplasmic reticulum (ER) - eukaryotic lipid synthesis. *De novo* synthesis of fatty acids occurs inside the chloroplast and can follow two pathways: (i) prokaryotic pathway: fatty acids are transported to envelope membranes and used for glycolipid synthesis (galactolipids and sulfolipids); (ii) eukaryotic pathway: fatty acids are exported as free fatty acids to envelope membranes and their corresponding CoA thioesters are transferred to the ER to integrate lipid structure. Lipid precursors assembled at the ER are exported to the outer envelope membrane for glycolipid synthesis. Glycolipids are incorporated into thylakoid membranes. Although lipid trafficking between membranes occurs through unknown mechanisms (dash lines), it is believed that transport between the ER and the outer envelope membrane occurs by direct contact sites between both membranes or via vesicles; while transport between inner envelope membrane and thylakoid membrane is mediated by vesicles. Under phosphate starvation (Pi), DGDG can be transported to ER and then to the plasma membrane to replace phospholipids. Figure adapted from Dörmann^19^.

Endosymbiosis is a rare process in nature because it requires the integration of host and symbiont membranes and their common evolution^12^. While kleptoplasty is a general phenomenon in protists^13, 14^, to date, sacoglossans are the only metazoans known to maintain this type of association^15^. This mechanism of endosymbiosis could have some similar features to the interaction between eukaryotic cells and endosymbiotic cyanobacteria, which gave rise to chloroplast-containing eukaryotes^12^. Despite the evolution from cyanobacteria to vascular plants, molecular composition of chloroplasts has been highly conserved^16^. Chloroplast membranes are characterized by the occurrence of high proportions of glycolipids, being monogalactosyl diacylglycerol (MGDG) and digalactosyl diacylglycerol (DGDG) the most abundant^16^, and sulfolipids (sulfoquinovosyl diacylglycerol, SQDG), which are not found in extraplastidial membranes. These classes of specific lipids are biosynthesized within the chloroplast^17–19^ (Fig. 1). A conserved proportion of these molecules integrates the complexes of photosynthetic machinery of chloroplasts, being critical to sustain an optimal rate of photosynthesis^20^. Inner chloroplast envelope and thylakoid membranes show similar relative proportions of lipids, highlighting the role of the former in the biogenesis of thylakoid membranes^20^. Additionally, betaine lipids (diacylglyceryl*-N,N,N-*trimethyl homoserine, DGTS) are a class of lipids found in algae and that can be translocated to the chloroplast^21^. DGTS is considered as a more ancient membrane lipid, which has been progressively replaced by phosphatidylcholine (PC) during the evolution of vascular plants^22^. Although algae have preserved the DGTS biosynthetic pathway^23^, DGTS competes with PC and therefore the levels of both lipid classes are reciprocal^22^.

Lipidomic analyses applied to marine samples can provide information at the molecular level, being successfully used to explain biological and molecular processes^24–26^. In this sense, we selected the ecological model *E. viridis* (Montagu, 1804) and *Codium tomentosum* (Stackhouse, 1797) to investigate the association of functional macroalgal chloroplasts inside animal cells, by using lipidomic tools. *Elysia viridis* collected in the coast of Portugal has a narrow feeding preference, mostly retaining functional plastids from genus *Codium* ^27^, thus limiting the origin of plastid lipids. Furthermore, *E. viridis* displays a relatively long-term retention of kleptoplasts^3, 4^, which allows researchers to perform experiments on individuals that already display well established functional kleptoplasts inside their animal cells.

Glycolipids and betaine lipids isolated from total lipid extracts of *E. viridis* and *C. tomentosum* were studied using hydrophilic interaction liquid chromatography-mass spectrometry (HILIC-LC-MS). The objective of the present study was to investigate if kleptoplasty promoted any major shifts in the lipidome of *C. tomentosum* plastids sequestered by the sacoglossan sea slug *E. viridis*. Hence, we focused the analysis on lipid classes known to be exclusive of chloroplast membranes (i.e., glycolipids), as well as on betaine lipids.

## Results

HILIC-LC-MS and MS/MS allowed the identification of glycolipids (in fraction 3, see Methods) and betaine lipids (in fraction 4) in the lipid extracts obtained from the marine species *C. tomentosum* and *E. viridis*. The information gathered with the high resolution HILIC-LC-MS and MS/MS analyses provided the detailed structural information to identify the different lipid classes and their molecular species profiles (see Supplementary Table S1). Overall, seventy-six molecular species were identified in *C. tomentosum* and sixty-two molecular species in *E. viridis* samples, as glycolipids and betaine lipids.

### Profile of glycolipids

Glycolipids were identified in fraction 3 for both *C. tomentosum* and *E. viridis*, distributed between galactolipid (MGDG and DGDG) and sulfolipid classes. Twenty-five molecular species were identified in *C. tomentosum* and twenty-one molecular species in *E. viridis* samples.

### Galactolipids

**Figure 2:**
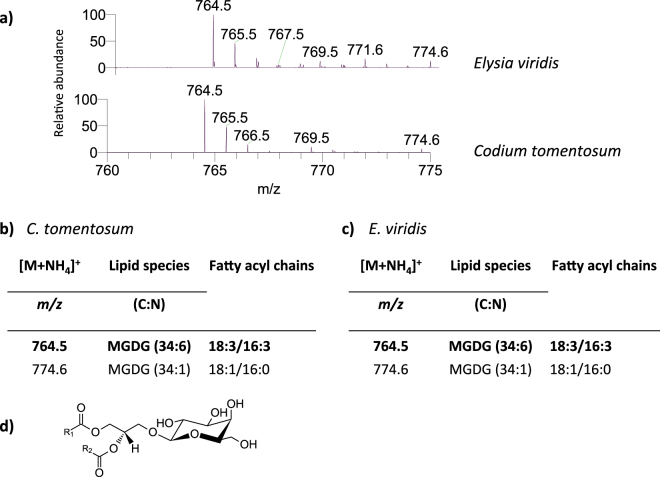
Lipidomic profile of monogalactosyl diacylglycerol (MGDG) in *Codium tomentosum* and *Elysia viridis*. (**a**) HILIC-LC-MS spectra of MGDG molecular species detected in *C. tomentosum* and *E. viridis* samples and identified as [M + NH_4_]^+^ ions; (**b**) Molecular species of MGDG identified in *C. tomentosum* samples (C represents the total number of carbon atoms and N the total number of double bonds on the fatty acyl chains; bold *m/z* value corresponds to the most abundant molecular species detected in HILIC-LC-MS spectrum); (**c**) Molecular species of MGDG identified in *E. viridis* samples; (**d**) General structure of MGDG.

**Figure 3:**
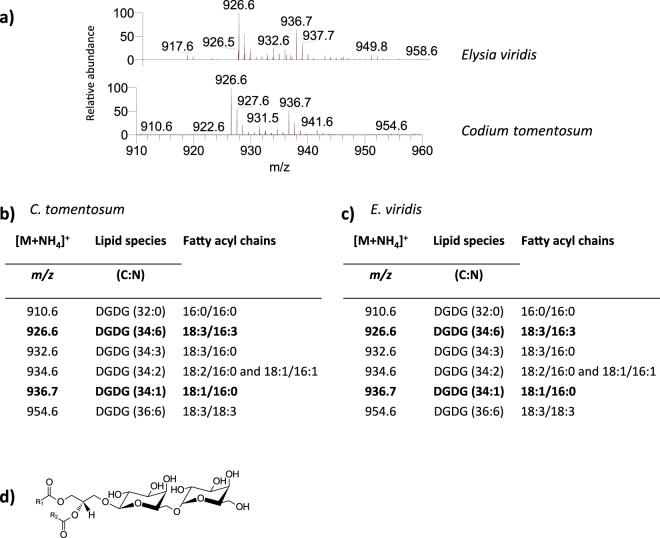
Lipidomic profile of digalactosyl diacylglycerol (DGDG) in *Codium tomentosum* and *Elysia viridis*. (**a**) HILIC-LC-MS spectra of DGDG molecular species detected in *C. tomentosum* and *E. viridis* samples and identified as [M + NH_4_]^+^ ions; (**b**) Molecular species of DGDG identified in *C. tomentosum* samples (C represents the total number of carbon atoms and N the total number of double bonds on the fatty acyl chains; bold *m/z* values correspond to the most abundant molecular species detected in HILIC-LC-MS spectrum); (**c**) Molecular species of DGDG identified in *E. viridis* samples; (**d**) General structure of DGDG.

Two classes of galactolipids were identified in both marine organisms: MGDG (Fig. 2) and DGDG (Fig. 3). MGDG and DGDG were identified in HILIC-LC-MS spectra in positive mode as [M + NH_4_]^+^ ions^25^. Two molecular species of MGDG were identified in *C. tomentosum* and *E. viridis* samples: MGDG (18:3/16:3) and MGDG (18:1/16:0), corresponding to [M + NH_4_]^+^ ions at *m/z* 764.5 and 774.6, respectively (Fig. 2). Regarding DGDG, a total of six molecular species were identified in both marine organisms. The most abundant DGDG molecular species were DGDG (18:3/16:3) and DGDG (18:1/16:0) in both *C. tomentosum* and *E. viridis*, corresponding to [M + NH_4_]^+^ ions at *m/z* 926.6 and 936.7, respectively (Fig. 3).

### Sulfolipids

**Figure 4:**
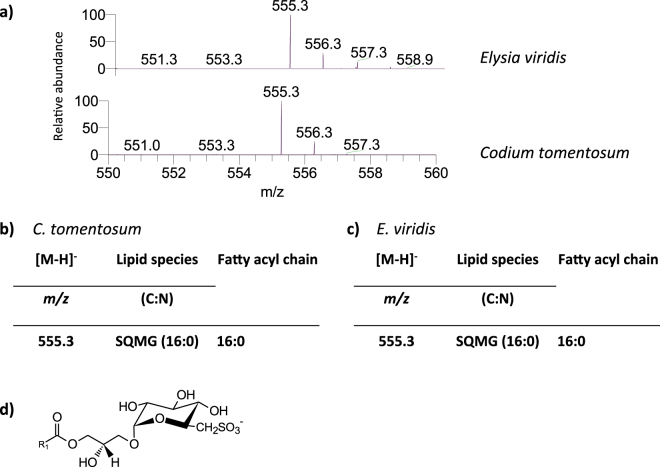
Lipidomic profile of sulfoquinovosyl monoacylglycerol (SQMG) in *Codium tomentosum* and *Elysia viridis*. (**a**) HILIC-LC-MS spectra of SQMG molecular species detected in *C. tomentosum* and *E. viridis* samples and identified as [M − H]^−^ ion; (**b**) Molecular species of SQMG identified in *C. tomentosum* samples (C represents the total number of carbon atoms and N the total number of double bonds on the fatty acyl chain); (**c**) Molecular species of SQMG identified in *E. viridis* samples; (**d**) General structure of SQMG.

**Figure 5:**
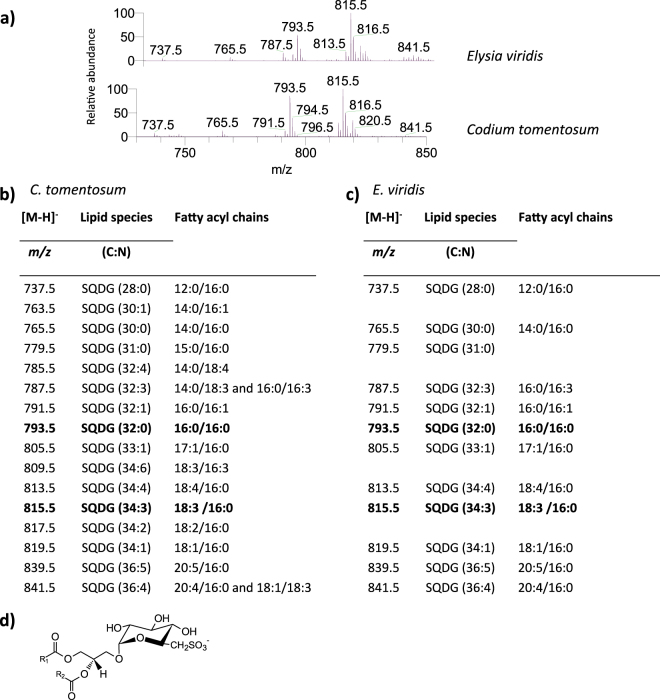
Lipidomic profile of sulfoquinovosyl diacylglycerol (SQDG) in *Codium tomentosum* and *Elysia viridis*. (**a**) HILIC-LC-MS spectra of SQDG molecular species detected in *C. tomentosum* and *E. viridis* samples and identified as [M − H]^−^ ions; (**b**) Molecular species of SQDG identified in *C*. *tomentosum* samples (C represents the total number of carbon atoms and N the total number of double bonds on the fatty acyl chains; bold *m/z* values correspond to the most abundant molecular species detected in HILIC-LC-MS spectrum); (**c**) Molecular species of SQDG identified in *E. viridis* samples; (**d**) General structure of SQDG.

Two classes of sulfolipids were identified in *C. tomentosum* and *E. viridis* samples: sulfoquinovosyl monoacylglycerol (SQMG) (Fig. 4) and SQDG (Fig. 5). Both classes were identified by HILIC-LC-MS in negative mode, by the observation of the [M − H]^−^ ions^25, 26^. In both marine organisms, only one SQMG molecular species was identified, the SQMG (16:0), corresponding to [M − H]^−^ ion at *m/z* 555.3 (Fig. 4).

Overall, sixteen molecular species of SQDG were identified in *C. tomentosum* samples, while in *E. viridis* samples twelve molecular species were identified. The same most abundant molecular species of SQDG were observed in both marine organisms, namely SQDG (18:3/16:0) and SQDG (16:0/16:0), corresponding to [M − H]^−^ ions at *m/z* 815.5 and 793.5, respectively (Fig. 5). The molecular species present only in *C. tomentosum* samples were: SQDG (14:0/16:1), SQDG (14:0/18:4), SQDG (18:3/16:3) and SQDG (18:2/16:0), corresponding to [M − H]^−^ ions at *m/z* 763.5, 785.5, 809.5 and 817.5, respectively (Fig. 5).

Glycolipid profile of *E. viridis* samples was validated through the survey of specimens placed under starvation, confirming the signature was from incorporated chloroplasts and not undigested algal material still present in the animal digestive system (see Supplementary Fig. S1 and Supplementary Table S2).

### Profile of betaine lipids

**Figure 6:**
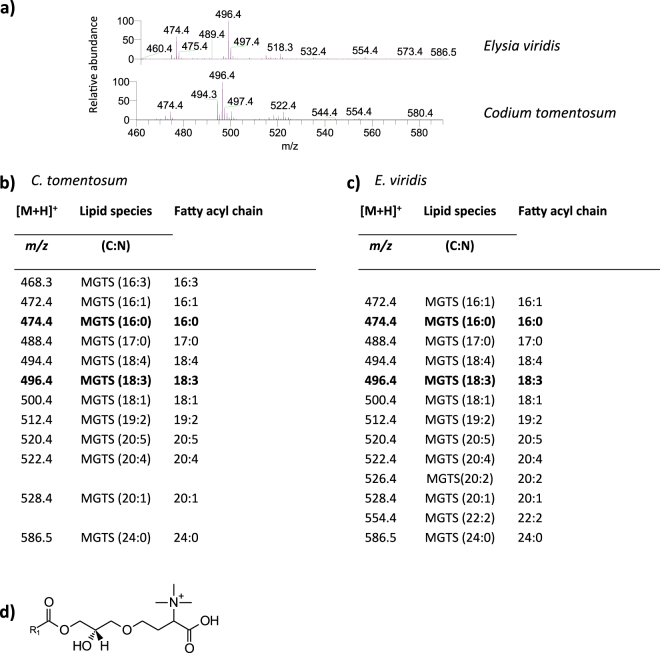
Lipidomic profile of monoacylglyceryl-*N,N,N*-trimethyl homoserine (MGTS) in *Codium tomentosum* and *Elysia viridis*. (**a**) HILIC-LC-MS spectra of MGTS molecular species detected in *C. tomentosum* and *E. viridis* samples and identified as [M + H]^+^ ions; (**b**) Molecular species of MGTS identified in *C. tomentosum* samples (C represents the total number of carbon atoms and N the total number of double bonds on the fatty acyl chain; bold *m/z* values correspond to the most abundant molecular species detected in HILIC-LC-MS spectrum); (**c**) Molecular species of MGTS identified in *E. viridis* samples; (**d**) General structure of MGTS.

**Figure 7:**
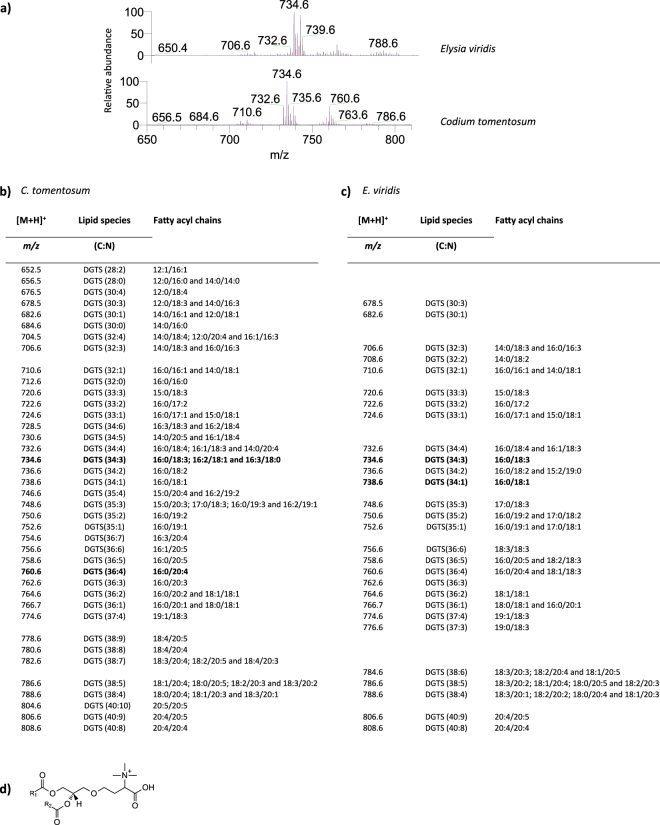
Lipidomic profile of diacylglyceryl*-N,N,N-*trimethyl homoserine (DGTS) in *Codium tomentosum* and *Elysia viridis*. (**a**) HILIC-LC-MS spectra of DGTS molecular species detected in *C. tomentosum* and *E. viridis* samples and identified as [M + H]^+^ ions; (**b**) Molecular species of DGTS identified in *C. tomentosum* samples (C represents the total number of carbon atoms and N the total number of double bonds on the fatty acyl chains; bold *m/z* values correspond to the most abundant molecular species detected in HILIC-LC-MS spectrum); (**c**) Molecular species of DGTS identified in *E. viridis* samples; (**d**) General structure of DGTS.

The analysis of the fraction 4, rich in betaine lipids and also containing phospholipids, provided the identification of two classes within betaine lipids in *C. tomentosum* and *E. viridis* samples: monoacylglyceryl-*N,N,N*-trimethyl homoserine (MGTS) (Fig. 6) and DGTS (Fig. 7). Both classes were identified as positive [M + H]^+^ ions^25, 26^. The analysis of MS/MS spectra allowed the identification of twelve molecular species of MGTS in *C. tomentosum* samples and thirteen in *E. viridis* samples. The most abundant molecular species were MGTS (18:3) and MGTS (16:0) in both marine organisms, corresponding to [M + H]^+^ ions at *m/z* 496.4 and 474.4, respectively (Fig. 6). *Codium tomentosum* samples showed the molecular species MGTS (16:3) in its lipidomic profile, corresponding to [M + H]^+^ ion at *m/z* 468.3, which was absent in *E. viridis* samples. On the other hand, two molecular species were identified *E. viridis* samples but not in *C. tomentosum* samples: MGTS (20:2) and MGTS (22:2), corresponding to [M + H]^+^ ions at *m/z* 526.4 and 554.4, respectively (Fig. 6).

Concerning the analysis of DGTS, a total of thirty-nine molecular species were identified in *C. tomentosum* and twenty-eight in *E. viridis* samples. The most abundant molecular species in *C. tomentosum* were DGTS (16:0/18:3), with minor contributions of DGTS (16:2/18:1), DGTS (16:3/18:0), and DGTS (16:0/20:4), the first three corresponding to [M + H]^+^ ions at *m/z* 734.6 and the latter to 760.6 (Fig. 7). In *E. viridis*, the most abundant molecular species were DGTS (16:0/18:3) and DGTS (16:0/18:1), corresponding to [M + H]^+^ ions at *m/z* 734.6 and 738.6, respectively (Fig. 7). Moreover, DGTS lipidomic profile showed fourteen molecular species that were only identified in *C. tomentosum* samples, while three DGTS molecular species were only identified in *E. viridis* samples (Fig. 7). This profile of betaine lipids was confirmed in *E. viridis* samples being exposed to starvation conditions (see Supplementary Fig. S1 and Supplementary Table S2).

## Discussion

The lipidome of chloroplast membranes of *C. tomentosum* was reflected in the sea slug *E. viridis*. Indeed, the most abundant lipid classes in chloroplast membranes, MGDG, DGDG and SQDG presented similar lipidomic profiles in *C. tomentosum* and *E. viridis* samples. The presence of these exclusive lipid classes of chloroplast membranes in *E. viridis* indicates that there are no major shifts in the lipidome promoted by kleptoplasty and suggests that the mechanisms necessary to perform photosynthesis are preserved during the process of endosymbiosis. This finding confirms the robustness of *C. tomentosum* chloroplasts, which do not experience major changes throughout ingestion and establishment in the digestive epithelial cells of the host^28^.

The lipidomic profile of *C. tomentosum* is in concordance with that previously described by da Costa *et al*.^25^. The glycolipids MGDG and DGDG are known to occur in all organisms performing oxygenic photosynthesis^16^. They are synthesized in the chloroplast envelope membranes and redistributed to the thylakoid membranes^19^, where, along with phosphatidylglycerol (PG), perform an important role in the stability and activity of photosynthetic complexes^16^. Moreover, MGDG and DGDG contribute to the stabilization of plastid membranes and lipid trafficking with extraplastidial membranes^20, 29^. Therefore, the conservation of MGDG and DGDG molecular species, along with fatty acyl composition, evidences the preservation of plastid membrane composition and function during kleptoplasty.

The lipidomic profile of sulfolipids shows the absence of several molecular species of SQDG in *E. viridis* when compared to *C. tomentosum* samples. These differences may be due to either extremely low concentration, likely below the detection limits, in sea slug samples or replacement during the process of endosymbiosis. Nevertheless, the most abundant molecular species were the same in both marine organisms. SQDG plays an important role in chloroplast development and regeneration^30^, thus the loss of these molecular species may be related with the process of establishment of the stolen plastid in cells of its new host. Although SQDG is associated with photosystem II^31^, the presence of SQDG does not always correlate with photosynthetic capacity^32^. However, SQDG performs relevant functions in chloroplasts and recently it has been discovered that in microalgae cells SQDG plays the role of sulphur storage lipids that is used for protein synthesis in early phases of sulphur deprivation^33^. On the other hand, under phosphate-limited conditions, phospholipids are replaced with non-phosphate containing lipids such as MGDG, DGDG and SQDG^32^. Under phosphate deprivation, DGDG levels increase and its excess is transferred to extraplastidial membranes to replace phospholipids (Fig. 1), limiting the consumption of phosphate for membrane lipid synthesis^32^. Furthermore, since SQDG and PG are anionic lipids, SQDG partially replaces PG to maintain the anionic surface charge of thylakoid membranes^32^. Although glycolipids can replace phospholipids under phosphate deprivation, lipid trafficking with extraplastidial membranes is limited to DGDG, since SQDG and MGDG were never detected outside plastids^34, 35^.

The location of glycolipids in the membrane of chloroplasts and thylakoids has been related with signalling and coordination functions, regulating chloroplast lipids and cytosolic partners^16, 20^. Since the glycolipid profile was preserved in kleptoplasts, these functions are likely retained during the process of chloroplast sequestration in sea slug cells.

MGTS and DGTS belong to a less studied class of lipids, whose metabolic pathways and functions are still not well characterized in marine algae^36^. DGTS has been substituted by PC during the evolution of vascular plants^37^, thus their distribution is limited to algae, lower plants and fungi^22^. A study in freshwater algae has suggested that DGTS acts as a donor of diacylglycerol (DAG), as well as polyunsaturated fatty acids, which will be used in the biosynthesis of galactolipids^38^. In accordance, rhythmic fluctuation of DGTS with time was inversely correlated with the levels of MGDG^39^. The similar fatty acyl composition in MGDG and DGTS, in both *C. tomentosum* and *E. viridis*, supports the hypothesis that DGTS acts as a donor of DAG^38, 39^. Our results corroborate that the fatty acyl composition of MGDG molecular species identified in *C. tomentosum* samples are present in DGTS molecular species (i.e., MGDG (18:3/16:3) and MGDG (18:1/16:0) versus DGTS (18:3/16:3) and DGTS (18:1/16:0)). However, in *E. viridis* samples, the molecular species DGTS (18:1/16:0) appears as one of the most abundant in this lipid class. This suggests that the metabolic pathways in which DGTS (18:1/16:0) is used as donor of DAG for the synthesis of MGDG (18:1/16:0) may not be as efficient when chloroplasts are sequestered by *E. viridis* cells, promoting an increase of this DGTS molecular species in kleptoplasts. On the other hand, the differences in the lipidomic profile of DGTS between the macroalgae and the sea slug point towards the occurrence of a minor remodelling during the process of endosymbiosis. Interestingly, some SQDG and DGTS molecular species identified in *C. tomentosum*, but not in *E. viridis* samples, display a similar fatty acyl composition. The absence of these molecular species in *E. viridis* may be related to chloroplast membrane adjustments during the integration in their new host cells^11^, or, as it was suggested above, to an extremely low concentration of these molecules in the sea slug.

The lipidomic approach used herein has opened a new perspective in the study of kleptoplasty. The minor differences recorded in the lipidome of plastid membranes in *C. tomentosum* and *E. viridis* indicate that the integrity of chloroplast membranes is conserved during the process of endosymbiosis.

## Methods

### Sampling

Samples of *C. tomentosum* and specimens of *E. viridis* were collected in September 2015 on the intertidal rocky shore of Praia de Labruge (41°16′28.9″N; 8°43′45.3″W), Vila do Conde (Portugal). Macroalga and sea slug samples were rinsed with freshwater purified through reverse osmosis, frozen, freeze-dried and stored individually at −20 °C for biochemical analysis. A total of five samples of *C. tomentosum* (n = 5) and *E. viridis* (n = 5) were analysed individually. Additionally, four specimens of *E. viridis* (n = 4) were placed under starvation during one week (a time frame that allows sea slugs to completely empty their guts) and subsequently analysed individually. The analysis of starved sea slugs was performed to validate the results presented in this study, as any algal lipid species detected in these specimens could only originate from kleptoplasts.

### Lipid extraction

The Bligh and Dyer method^40^ was used to extract total lipids from *C. tomentosum* and *E. viridis* samples. *Codium tomentosum* samples were previously macerated with liquid nitrogen. Briefly, samples were mixed with 5 mL (*C. tomentosum*)/600 µL (*E. viridis*) of methanol in glass centrifuge tubes, vortexed, followed by the addition of 2.5 mL (*C. tomentosum*)/300 µL (*E. viridis*) of chloroform and vortexing. Then the samples were incubated on ice for 3 h (*C. tomentosum*)/30 min (*E. viridis*). An additional volume of 3 mL (*C. tomentosum*) of methanol:chloroform (2:1, *v*/*v*)/300 µL (*E. viridis*) of chloroform was added, along with 1.3 mL (*C. tomentosum*)/300 µL (*E. viridis*) of ultrapure water. Following vigorous homogenization, samples were centrifuged at 2000 rpm for 10 min at room temperature to resolve a two-phase system. The lipids were recovered from the organic lower phase. The extraction was repeated twice. The organic phases were dried under a nitrogen stream and preserved at −20 °C for further analysis.

### Fractionation of lipid extract

Lipid extracts were fractionated according to da Costa *et al*. method^41^. Fractionation was performed by solid-phase extraction using a Supelclean™ LC–Si SPE Tube (bed wt. 500 mg, volume 3 mL cartridges, SUPELCO). Column was activated with 6 mL of *n*-hexane and then the sample of lipid extract (110 and 4 mg of macroalgae and sea slug, respectively) was applied after to be dissolved on 300 μL of chloroform. Subsequently, the following sequential elution was performed to separate lipid fractions: 5 mL (*C. tomentosum*)/4 mL (*E. viridis*) of chloroform, 8 mL (*C. tomentosum*)/3 mL (*E. viridis*) of diethyl ether:acetic acid (98:2, *v*/*v*), 5 mL of acetone:methanol (9:1, *v*/*v*) and 6 mL of methanol. Total lipid extracts were fractionated in four lipid fractions: fraction 1 (rich in neutral lipids), fraction 2 (rich in pigments), fraction 3 (rich in glycolipids) and fraction 4 (rich in betaine lipids and phospholipids). Fractions 3 and 4 were recovered separated and dried under nitrogen stream and stored at −20 °C prior to analysis by HILIC-LC-MS.

### Hydrophilic interaction liquid chromatography–mass spectrometry (HILIC-LC-MS)

A high performance LC (HPLC) system (Thermo scientific Accela^TM^) with an autosampler coupled online to the Q-Exactive® mass spectrometer with Orbitrap® technology was used. The solvent system consisted of two mobile phases as follows: mobile phase A (acetonitrile:methanol:water 50:25:25, *v*/*v*/*v* with 1 mM ammonium acetate) and mobile phase B (acetonitrile:methanol 60:40, *v*/*v*, with 1 mM ammonium acetate). Initially, 0% of mobile phase A was held isocratically for 8 min, followed by a linear increase to 60% of A within 7 min and a maintenance period of 15 min, returning to the initial conditions in 10 min. A volume of 5 µL of each sample containing 5 µg of lipid extract and 95 µL of eluent B was introduced into the Ascentis® Si column (15 cm × 1 mm, 3 µm, Sigma-Aldrich) with a flow rate of 40 µL min^−1^ and at 30 °C. The mass spectrometer with Orbitrap® technology was operated in simultaneous positive (electrospray voltage 3.0 kV) and negative (electrospray voltage −2.7 kV) modes with high resolution with 70.000 and automatic gain control (AGC) target of 1e6, the capillary temperature was 250 °C and the sheath gas flow was 15 U. In MS/MS experiments, a resolution of 17.500 and AGC target of 1e5 was used and the cycles consisted in one full scan mass spectrum and ten data-dependent MS/MS scans were repeated continuously throughout the experiments with the dynamic exclusion of 60 seconds and intensity threshold of 1e4. Normalized collision energy™ (CE) ranged between 25, 30 and 35 eV. Data acquisition was carried out using the Xcalibur data system (V3.3, Thermo Fisher Scientific, USA). All the analyses were performed in analytical triplicates. The identification of molecular species of polar lipids was based on the assignment of the molecular ions observed in LC-MS/MS spectra. A mass accuracy (Qual Browser) of ≤5 ppm was used in order to uniquely identify the molecular species.

## Electronic supplementary material

Supplementary Material
