# Supplementary material for: Kleptoplasty does not promote major shifts in the lipidome of macroalgal chloroplasts sequestered by the sacoglossan sea slug Elysia viridis
Source: Sci Rep. 2017 Sep 13;7:11502. doi: 10.1038/s41598-017-12008-z (PMC5597624; doi:10.1038/s41598-017-12008-z)
Supplement: Supplementary file 1 — Supplementary Material [file 41598_2017_12008_MOESM1_ESM.pdf]

Kleptoplasty does not promote major shifts in the lipidome of macroalgal chloroplasts sequestered by the sacoglossan sea slug *Elysia viridis*

Felisa Rey[1*], Elisabete da Costa[2], Ana M. Campos[2], Paulo Cartaxana[1], Elisabete Maciel[1,2], Pedro Domingues[2], M. Rosário M. Domingues[2], Ricardo Calado[1] & Sónia Cruz[1*]

[1] Departamento de Biologia & CESAM, Universidade de Aveiro, Campus Universitário de Santiago, 3810-193 Aveiro, Portugal.

[2] Centro de Espetrometria de Massa, Departmento de Química & QOPNA, Universidade de Aveiro, Campus Universitário de Santiago, 3810-193 Aveiro, Portugal.

*Corresponding authors:
F.R. (email: felisa.rey@gmail.com)
S.C. (email: sonia.cruz@ua.pt)

Supplementary Table S1

Lipidomic profile of glycolipids and betaine lipids of *Codium tomentosum* and *Elysia viridis* samples.
Total molecular species identified in *C. tomentosum* and *E. viridis* samples, as glycolipids and betaine lipids, by mass accuracy HILIC−LC−MS and MS/MS analyses.
C represents the total number of carbon atoms and N the total number of double bonds on the fatty acyl chains.

Formula		Sample				Sample			
		Codium tomentosum				*Elysia viridis*			
Galactolipids $[M+NH4]^+$	*m/z* library	*m/z* experiment	Lipid species (C:N)	Fatty acyl chain	Delta ppm	*m/z* experiment	Lipid species (C:N)	Fatty acyl chain	Delta ppm
C43H74O10N	764.531273	764.52911	MGDG (34:6)	18:3/16:3	-2.111	764.52021	MGDG(34:6)	18:3/16:3	-0.672
C43H84O10N	774.609524	774.60742	MGDG (34:1)	18:1/16:0	-2.006	774.60875	MGDG(34:1)	18:1/16:0	-0.224
Galactolipids $[M+NH4]^+$	*m/z* library	*m/z* experiment	Lipid species (C:N)	Fatty acyl chain	Delta ppm	*m/z* experiment	Lipid species (C:N)	Fatty acyl chain	Delta ppm
C47H92O15N	910.646699	910.64390	DGDG (32:0)	16:0/16:0	-2.523	910.64675	DGDG (32:0)	16:0/16:0	0.662
C49H84O15N	926.584099	926.58181	DGDG (34:6)	18:3/16:3	-1.875	926.58269	DGDG (34:6)	18:3/16:3	-0.925
C49H90O15N	932.631049	932.62730	DGDG (34:3)	18:3/16:0	-3.450	932.62900	DGDG (34:3)	18:3/16:0	-1.605
C49H92O15N	934.646699	934.64350	DGDG (34:2)	18:2/16:0 and 18:1/16:1	-2.822	934.64433	DGDG (34:2)	18:2/16:0 and 18:1/16:1	-1.944
C49H94O15N	936.662349	936.65925	DGDG (34:1)	18:1/16:0	-2.720	936.66056	DGDG (34:1)	18:1/16:0	-1.321
C51H88O15N	954.615399	954.61264	DGDG (36:6)	18:3/18:3	-2.312	954.61408	DGDG (36:6)	18:3/18:3	-0.804
Sulfolipids $[M-H]^-$	*m/z* library	*m/z* experiment	Lipid species (C:N)	Fatty acyl chain	Delta ppm	*m/z* experiment	Lipid species (C:N)	Fatty acyl chain	Delta ppm
C25H47O11S	555.283912	555.28519	SQMG (16:0)	16:0	3.297	555.28668	SQMG (16:0)	16:0	3.783
Sulfolipids $[M-H]^-$	*m/z* library	*m/z* experiment	Lipid species (C:N)	Fatty acyl chain	Delta ppm	*m/z* experiment	Lipid species (C:N)	Fatty acyl chain	Delta ppm
C37H69O12S	737.450977	737.45256	SQDG (28:0)	12:0/16:0	2.896	737.45312	SQDG (28:0)	12:0/16:0	3.655
C39H71O12S	763.466627	763.46680	SQDG (30:1)	14:0/16:1	0.950				
C39H73O12S	765.482277	765.48370	SQDG (30:0)	14:0/16:0	2.580	765.48558	SQDG (30:0)	14:0/16:0	4.971
C40H75O12S	779.497927	779.49958	SQDG (31:0)	15:0/16:0	2.829	779.50003	SQDG (31:0)		3.791
C41H69O12S	785.450977	785.45206	SQDG (32:4)	14:0/18:4	2.082				
C41H71O12S	787.466627	787.46797	SQDG (32:3)	14:0/18:3 and 16:0/16:3	2.407	787.46832	SQDG (32:3)	16:0/16:3	2.850
C41H75O12S	791.497927	791.49958	SQDG (32:1)	16:0/16:1	2.786	791.50177	SQDG (32:1)	16:0/16:1	4.896
C41H77O12S	793.513576	793.51524	SQDG (32:0)	16:0/16:0	2.792	793.51748	SQDG (32:0)	16:0/16:0	4.934
C42H77O12S	805.513576	805.51539	SQDG (33:1)	17:1/16:0	2.936	805.51738	SQDG (33:1)	17:1/16:0	5.369
C43H69O12S	809.450977	809.45241	SQDG (34:6)	18:3/16:3	2.453				
C43H73O12S	813.482277	813.48389	SQDG (34:4)	18:4/16:0	2.662	813.48627	SQDG (34:4)	18:4/16:0	4.961
C43H75O12S	815.497927	815.49972	SQDG (34:3)	18:3/16:0	2.876	815.50166	SQDG (34:3)	18:3/16:0	5.255
C43H77O12S	817.513576	817.50987	SQDG (34:2)	18:2/16:0	-3.859				
C43H79O12S	819.529227	819.53104	SQDG (34:1)	18:1/16:0	2.886	819.53335	SQDG (34:1)	18:1/16:0	5.180
C45H75O12S	839.497927	839.49888	SQDG (36:5)	20:5/16:0	1.793	839.50108	SQDG (36:5)	20:5/16:0	4.414
C45H77O12S	841.513576	841.51472	SQDG (36:4)	20:4/16:0 and 18:1/18:3	2.014	841.51722	SQDG (36:4)	20:4/16:0	2.977

Formula		Sample				Sample			
		Codium tomentosum				*Elysia viridis*			
**Betaine lipids $[M+H]^+$**	***m/z* library**	***m/z* experiment**	**Lipid species (C:N)**	**Fatty acyl chain**	**Delta ppm**	***m/z* experiment**	**Lipid species (C:N)**	**Fatty acyl chain**	**Delta ppm**
C26H46O6N	468.332514	468.33046	MGTS (16:3)	16:3	-1.141				
C26H50O6N	472.363814	472.36316	MGTS (16:1)	16:1	-0.222	472.36220	MGTS (16:1)	16:1	-2.254
C26H52O6N	474.379464	474.37904	MGTS (16:0)	16:0	0.264	474.37785	MGTS (16:0)	16:0	-2.245
C27H54O6N	488.395114	488.39477	MGTS (17:0)	17:0	0.420	488.39374	MGTS (17:0)	17:0	-1.689
C28H48O6N	494.348164	494.34765	MGTS (18:4)	18:4	0.072	494.34628	MGTS (18:4)	18:4	-2.700
C28H50O6N	496.363814	496.36335	MGTS (18:3)	18:3	0.172	496.36224	MGTS (18:3)	18:3	-2.064
C28H54O6N	500.395114	500.39474	MGTS (18:1)	18:1	0.350	500.39360	MGTS (18:1)	18:1	-1.928
C29H54O6N	512.395114	512.39646	MGTS (19:2)	19:2	3.699	512.39423	MGTS (19:2)	19:2	-0.653
C30H50O6N	520.363814	520.36323	MGTS (20:5)	20:5	-0.067	520.36098	MGTS (20:5)	20:5	-4.391
C30H52O6N	522.379464	522.37902	MGTS (20:4)	20:4	0.201	522.37681	MGTS (20:4)	20:4	-4.029
C30H56O6N	526.410764					526.40950	MGTS (20:2)	20:2	-1.358
C30H58O6N	528.426414	528.42529	MGTS (20:1)	20:1	-1.088	528.42478	MGTS (20:1)	20:1	-2.053
C32H60O6N	554.442064					554.44060	MGTS (22:2)	22:2	-1.650
C34H68O6N	586.504664	586.50298	MGTS (24:0)	24:0	-1.936	586.50306	MGTS (24:0)	24:0	-1.799

**Betaine lipids $[M+H]^+$**	***m/z* library**	***m/z* experiment**	**Lipid species (C:N)**	**Fatty acyl chain**	**Delta ppm**	***m/z* experiment**	**Lipid species (C:N)**	**Fatty acyl chain**	**Delta ppm**
C38H70O7N	652.515229	652.51350	DGTS (28:2)	12:1/16:1	-1.181				
C38H74O7N	656.546529	656.54689	DGTS (28:0)	12:0/16:0 and 14:0/14:0	1.386				
C40H70O7N	676.515229	676.51417	DGTS (30:4)	12:0/18:4	-0.754				
C40H72O7N	678.530879	678.53032	DGTS (30:3)	12:0/18:3 and 14:0/16:3	-0.015	678.52914	DGTS (30:3)		-1.754
C40H76O7N	682.562179	682.56110	DGTS (30:1)	14:0/16:1 and 12:0/18:1	-0.762	682.55999	DGTS (30:1)		-2.403
C40H78O7N	684.577829	684.57742	DGTS (30:0)	14:0/16:0	0.204				
C42H74O7N	704.546529	704.54552	DGTS (32:4)	14:0/18:4; 12:0/20:4 and 16:1/16:3	-0.653				
C42H76O7N	706.562179	706.56125	DGTS (32:3)	14:0/18:3 and 16:0/16:3	-0.538	706.56039	DGTS (32:3)	14:0/18:3 and 16:0/16:3	-1.755
C42H78O7N	708.577829					708.57508	DGTS (32:2)	14:0/18:2	-3.105
C42H80O7N	710.593479	710.59233	DGTS (32:1)	16:0/16:1 and 14:0/18:1	-0.845	710.59113	DGTS (32:1)	14:0/18:1 and 16:0/16:1	-2.533
C42H82O7N	712.609128	712.60657	DGTS (32:0)	16:0/16:0	-2.821				
C43H78O7N	720.577829	720.57665	DGTS (33:3)	15:0/18:3	-0.874	720.57623	DGTS (33:3)	15:0/18:3	-1.457
C43H80O7N	722.593479	722.59231	DGTS (33:2)	16:0/17:2	-0.858	722.59073	DGTS (33:2)	15:0/18:2	-3.045
C43H82O7N	724.609128	724.60807	DGTS (33:1)	16:0/17:1 and 15:0/18:1	-0.704	724.60676	DGTS (33:1)	16:0/17:1 and 15:0/18:1	-2.512
C44H74O7N	728.546529	728.54598	DGTS (34:6)	16:3/18:3 and 16:2/18:4	0.000				
C44H76O7N	730.562179	730.56216	DGTS (34:5)	14:0/20:5; 16:1/18:4	0.725				
C44H78O7N	732.577829	732.57698	DGTS (34:4)	16:0/18:4; 16:1/18:3 and 14:0/20:4	-0.410	732.57729	DGTS (34:4)	16:0/18:4; 16:1/18:3	0.013
C44H80O7N	734.593479	734.59242	DGTS (34:3)	16:0/18:3; 16:2/18:1 and 16:3/18:0	-0.695	734.59135	DGTS (34:3)	16:0/18:3	-2.138
C44H82O7N	736.609128	736.60558	DGTS (34:2)	16:0/18:2	-4.073	736.60558	DGTS (34:2)	16:0/18:2 and 15:2/19:0	-4.073
C44H84O7N	738.624779	738.62311	DGTS (34:1)	16:0/18:1	-1.517	738.62187	DGTS (34:1)	16:0/18:1	-3.196
C45H80O7N	746.593479	746.59178	DGTS (35:4)	15:0/20:4 and 16:2/19:2	-1.541				
C45H82O7N	748.609128	748.57181	DGTS (35:3)	15:0/20:3; 17:0/18:3; 16:0/19:3 and 16:2/19:1	-0.514	748.60641	DGTS (35:3)	17:0/18:3	-2.899
C45H84O7N	750.624779	750.62242	DGTS (35:2)	16:0/19:2	-2.412	750.62073	DGTS (35:2)	16:0/19:2 and 17:0/18:2	-4.663
C45H86O7N	752.640429	752.63853	DGTS (35:1)	16:0/19:1	-1.794	752.63659	DGTS (35:1)	16:0/19:1 and 17:0/18:1	-4.372
C46H76O7N	754.562179	754.56053	DGTS (36:7)	16:3/20:4	-1.458				
C46H78O7N	756.577829	756.57553	DGTS (36:6)	16:1/20:5	-2.313	756.58068	DGTS (36:6)	18:3/18:3	4.494
C46H80O7N	758.593479	758.59192	DGTS (36:5)	16:0/20:5	-1.332	758.59225	DGTS (36:5)	16:0/20:5 and 18:2/18:3	-0.897
C46H82O7N	760.609128	760.60730	DGTS (36:4)	16:0/20:4	-1.683	760.60594	DGTS (36:4)	16:0/20:4 and 18:1/18:3	-3.471
C46H84O7N	762.624779	762.62066	DGTS (36:3)	16:0/20:3	-4.682	762.6212	DGTS (36:3)		-3.974
C46H86O7N	764.640429	764.63689	DGTS (36:2)	16:0/20:2 and 18:1/18:1	-3.911	764.63724	DGTS (36:2)	18:1/18:1	-3.453

Formula		Sample				Sample			
		Codium tomentosum				***Elysia viridis***			
**Betaine lipids [M+H]$^{+}$**	***m/z* library**	***m/z* experiment**	**Lipid species (C:N)**	**Fatty acyl chain**	**Delta ppm**	***m/z* experiment**	**Lipid species (C:N)**	**Fatty acyl chain**	**Delta ppm**
C46H88O7N	766.656079	766.65320	DGTS (36:1)	16:0/20:1 and 18:0/18:1	-3.040	766.65262	DGTS (36:1)	18:0/18:1; 16:0/20:1	-3.796
C47H84O7N	774.624779	774.62289	DGTS (37:4)	19:1/18:3	-1.730	774.62393	DGTS (37:4)	19:1/18:3	-0.388
C47H86O7N	776.640429					776.63693	DGTS (37:3)	19:0/18:3	-3.799
C48H76O7N	778.562179	778.55898	DGTS (38:9)	18:4/20:5	-3.404				
C48H78O7N	780.577829	780.57520	DGTS (38:8)	18:4/20:4	-2.665				
C48H80O7N	782.593479	782.59130	DGTS (38:7)	18:3/20:4; 18:2/20:5 and 18:4/20:3	-2.083				
C48H82O7N	784.609128					784.60859	DGTS (38:6)	18:3/20:3; 18:2/20:4 and 18:1/20:5	0.012
C48H84O7N	786.624779	786.62275	DGTS (38:5)	18:1/20:4; 18:0/20:5; 18:2/20:3 and 18:3/20:2	-1.882	786.62356	DGTS (38:5)	18:3/20:2; 18:2/20:3; 18:1/20:4 and 18:0/20:5	-0.852
C48H86O7N	788.640429	788.63804	DGTS (38:4)	18:0/20:4; 18:1/20:3 and 18:3/20:1	-2.334	788.63814	DGTS (38:4)	18:3/20:1; 18:2/20:2; 18:1/20:3 and 18:0/20:4	-2.207
C50H78O7N	804.577829	804.57514	DGTS (40:10)	20:5/20:5	-2.660				
C50H80O7N	806.593479	806.59169	DGTS (40:9)	20:4/20:5	-1.538	806.59151	DGTS (40:9)	20:4/20:5	-1.761
C50H82O7N	808.609128	808.60721	DGTS (40:8)	20:4/20:4	-1.695	808.60752	DGTS (40:8)	20:4/20:4	-1.311

Supplementary Figure S1. HILIC–LC–MS spectra of the molecular species of a) monogalactosyl diacylglycerol (MGDG) identified as $[M + NH_4]^+$ ions; b) digalactosyl diacylglycerol (DGDG) identified as $[M + NH_4]^+$; c) sulfoquinovosyl monoacylglycerol (SQMG) identified as $[M - H]^-$ ion; d) sulfoquinovosyl diacylglycerol (SQDG) identified as $[M - H]^-$ ions; e) monoacylglyceryl-*N,N,N*-trimethyl homoserine (MGTS) identified as $[M + H]^+$ ions and f) diacylglyceryl-*N,N,N*-trimethyl homoserine (DGTS) identified as $[M + H]^+$ ions, detected in specimens of *Elysia viridis* being exposed to starvation during one week.

a) MGDG

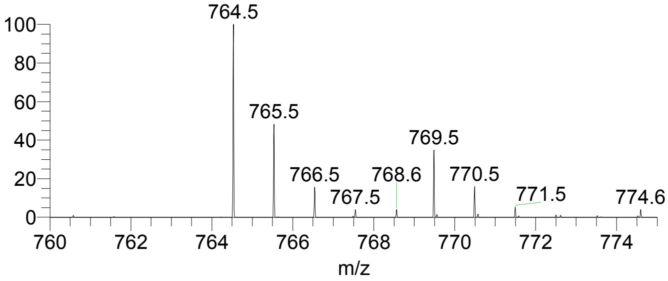

b) DGDG

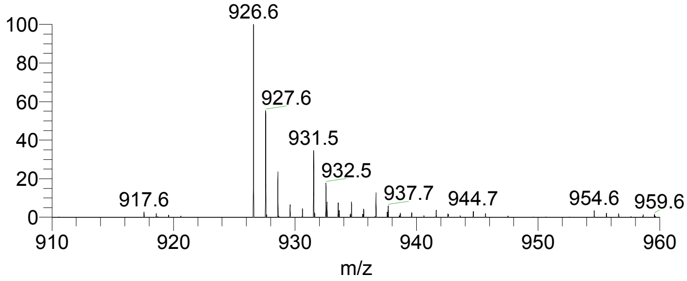

c) SQMG

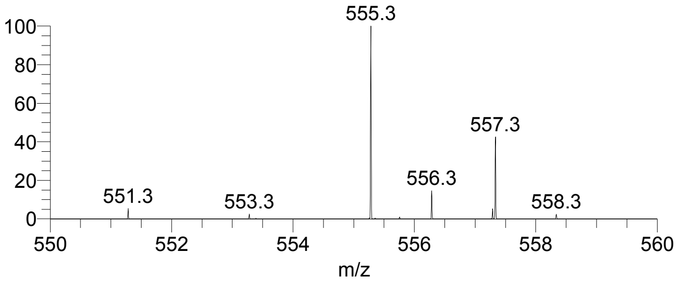

d) SQDG

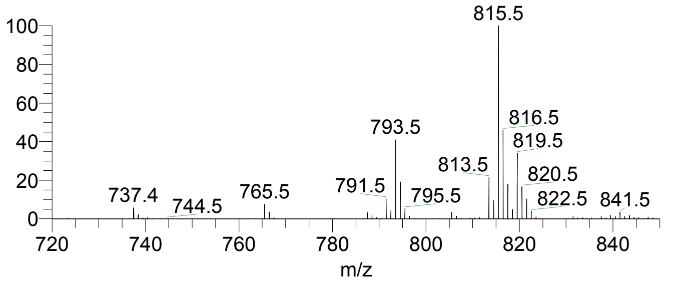

e) MGTS

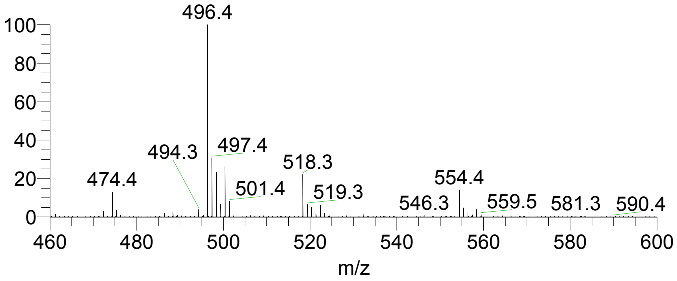

f) DGTS

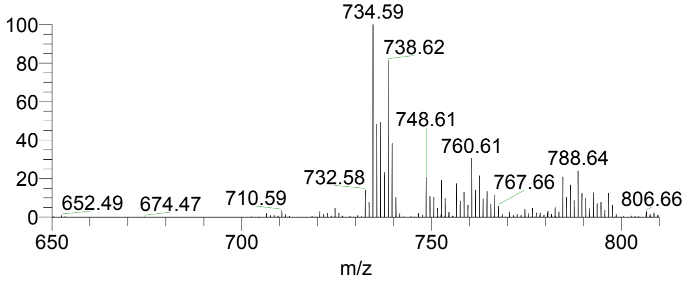

Supplementary Table S2

Lipidomic profile of glycolipids and betaine lipids of *Elysia viridis* and *E. viridis* being exposed to starvation during one week.
Total molecular species identified in *E. viridis* samples, as glycolipids and betaine lipids, by mass accuracy HILIC−LC−MS.
C represents the total number of carbon atoms and N the total number of double bonds on the fatty acyl chains.

Formula		Sample			Sample		
		Elysia viridis			*Elysia viridis* Starvation		
**Galactolipids [M+NH4]$^+$**	***m/z* library**	***m/z* experiment**	**Lipid species (C:N)**	**Delta ppm**	***m/z* experiment**	**Lipid species (C:N)**	**Delta ppm**
C43H74O10N	764.531273	764.52021	MGDG(34:6)	-0.672	764.53109	MGDG(34:6)	0.479
C43H84O10N	774.609524	774.60875	MGDG(34:1)	-0.224	774.60908	MGDG(34:1)	0.137
**Galactolipids [M+NH4]$^+$**	***m/z* library**	***m/z* experiment**	**Lipid species (C:N)**	**Delta ppm**	***m/z* experiment**	**Lipid species (C:N)**	**Delta ppm**
C47H92O15N	910.646699	910.64675	DGDG (32:0)	0.662	910.64434	DGDG (32:0)	-1.985
C49H84O15N	926.584099	926.58269	DGDG (34:6)	-0.925	926.58384	DGDG (34:6)	0.316
C49H90O15N	932.631049	932.62900	DGDG (34:3)	-1.605	932.62963	DGDG (34:3)	-0.930
C49H92O15N	934.646699	934.64433	DGDG (34:2)	-1.944	934.64442	DGDG (34:2)	-1.848
C49H94O15N	936.662349	936.66056	DGDG (34:1)	-1.321	936.66134	DGDG (34:1)	-0.488
C51H88O15N	954.615399	954.61408	DGDG (36:6)	-0.804	954.61569	DGDG (36:6)	0.883
**Sulfolipids [M-H]$^-$**	***m/z* library**	***m/z* experiment**	**Lipid species (C:N)**	**Delta ppm**	***m/z* experiment**	**Lipid species (C:N)**	**Delta ppm**
C25H47O11S	555.283912	555.28668	SQMG (16:0)	3.783	555.28238	SQMG (16:0)	-1.764
**Sulfolipids [M-H]$^-$**	***m/z* library**	***m/z* experiment**	**Lipid species (C:N)**	**Delta ppm**	***m/z* experiment**	**Lipid species (C:N)**	**Delta ppm**
C37H69O12S	737.450977	737.45312	SQDG (28:0)	3.655	737.44953	SQDG (28:0)	-1.213
C39H73O12S	765.482277	765.48558	SQDG (30:0)	4.971	765.48021	SQDG (30:0)	-1.979
C40H75O12S	779.497927	779.50003	SQDG (31:0)	3.791	779.49591	SQDG (31:0)	-1.879
C41H71O12S	787.466627	787.46832	SQDG (32:3)	2.850	787.46503	SQDG (32:3)	-1.327
C41H75O12S	791.497927	791.50177	SQDG (32:1)	4.896	791.49608	SQDG (32:1)	-1.636
C41H77O12S	793.513576	793.51748	SQDG (32:0)	4.934	793.51161	SQDG (32:0)	-1.783
C42H77O12S	805.513576	805.51738	SQDG (33:1)	5.369	805.51159	SQDG (33:1)	-1.781
C43H73O12S	813.482277	813.48627	SQDG (34:4)	4.961	813.48041	SQDG (34:4)	1.460
C43H75O12S	815.497927	815.50166	SQDG (34:3)	5.255	815.49572	SQDG (34:3)	-2.029
C43H79O12S	819.529227	819.53335	SQDG (34:1)	5.180	819.52734	SQDG (34:1)	-1.629
C45H75O12S	839.497927	839.50108	SQDG (36:5)	4.414	839.49502	SQDG (36:5)	-2.805
C45H77O12S	841.513576	841.51722	SQDG (36:4)	2.977	841.51172	SQDG (36:4)	-1.551
**Betaine lipids [M+H]$^+$**	***m/z* library**	***m/z* experiment**	**Lipid species (C:N)**	**Delta ppm**	***m/z* experiment**	**Lipid species (C:N)**	**Delta ppm**
C26H50O6N	472.363814	472.36220	MGTS (16:1)	-2.254	472.36309	MGTS (16:1)	-0.370
C26H52O6N	474.379464	474.37785	MGTS (16:0)	-2.245	474.37913	MGTS (16:0)	0.454
C27H54O6N	488.395114	488.39374	MGTS (17:0)	-1.689	488.39480	MGTS (17:0)	0.482
C28H48O6N	494.348164	494.34628	MGTS (18:4)	-2.700	494.34741	MGTS (18:4)	-0.414
C28H50O6N	496.363814	496.36224	MGTS (18:3)	-2.064	496.36332	MGTS (18:3)	0.111
C28H54O6N	500.395114	500.39360	MGTS (18:1)	-1.928	500.39477	MGTS (18:1)	0.410
C29H54O6N	512.395114	512.39423	MGTS (19:2)	-0.653	512.39906	MGTS (19:2)	4.495
C30H50O6N	520.363814	520.36098	MGTS (20:5)	-4.391	520.36245	MGTS (20:5)	-1.566
C30H52O6N	522.379464	522.37681	MGTS (20:4)	-4.029	522.37711	MGTS (20:4)	-3.455
C30H56O6N	526.410764	526.40950	MGTS (20:2)	-1.358	526.41074	MGTS (20:2)	0.998
C30H58O6N	528.426414	528.42478	MGTS (20:1)	-2.053	528.42591	MGTS (20:1)	0.085
C32H60O6N	554.442064	554.44060	MGTS (22:2)	-1.650	554.44161	MGTS (22:2)	0.171
C34H68O6N	586.504664	586.50306	MGTS (24:0)	-1.799	586.50381	MGTS (24:0)	-0.520
**Betaine lipids [M+H]$^+$**	***m/z* library**	***m/z* experiment**	**Lipid species (C:N)**	**Delta ppm**	***m/z* experiment**	**Lipid species (C:N)**	**Delta ppm**
C40H72O7N	678.530879	678.52914	DGTS (30:3)	-1.754	678.53098	DGTS (30:3)	0.958
C40H76O7N	682.562179	682.55999	DGTS (30:1)	-2.403	682.56178	DGTS (30:1)	0.220
C42H76O7N	706.562179	706.56039	DGTS (32:3)	-1.755	706.56222	DGTS (32:3)	0.835
C42H78O7N	708.577829	708.57508	DGTS (32:2)	-3.105	708.57631	DGTS (32:2)	-1.369
C42H80O7N	710.593479	710.59113	DGTS (32:1)	-2.533	710.59326	DGTS (32:1)	0.464
C43H78O7N	720.577829	720.57623	DGTS (33:3)	-1.457	720.57788	DGTS (33:3)	0.832
C43H80O7N	722.593479	722.59073	DGTS (33:2)	-3.045	722.59211	DGTS (33:2)	-1.135
C43H82O7N	724.609128	724.60676	DGTS (33:1)	-2.512	724.60894	DGTS (33:1)	0.496
C44H78O7N	732.577829	732.57729	DGTS (34:4)	0.013	732.57752	DGTS (34:4)	0.327
C44H80O7N	734.593479	734.59135	DGTS (34:3)	-2.138	734.59353	DGTS (34:3)	0.817
C44H82O7N	736.609128	736.60558	DGTS (34:2)	-4.073	736.60763	DGTS (34:2)	-1.290
C44H84O7N	738.624779	738.62187	DGTS (34:1)	-3.196	738.62412	DGTS (34:1)	-0.149
C45H82O7N	748.609128	748.60641	DGTS (35:3)	-2.899	748.60888	DGTS (35:3)	0.400
C45H84O7N	750.624779	750.62073	DGTS (35:2)	-4.663	750.62309	DGTS (35:2)	-1.519
C45H86O7N	752.640429	752.63659	DGTS (35:1)	-4.372	752.63990	DGTS (35:1)	0.026
C46H78O7N	756.577829	756.58068	DGTS (36:6)	4.494	752.63990	DGTS (36:6)	-2.393
C46H80O7N	758.593479	758.59225	DGTS (36:5)	-0.897	758.59143	DGTS (36:5)	-1.500
C46H82O7N	760.609128	760.60594	DGTS (36:4)	-3.471	760.60775	DGTS (36:4)	-1.092
C46H84O7N	762.624779	762.6212	DGTS (36:3)	-3.974	762.62403	DGTS (36:3)	-0.263
C46H86O7N	764.640429	764.63724	DGTS (36:2)	-3.453	764.64047	DGTS (36:2)	0.771
C46H88O7N	766.656079	766.65262	DGTS (36:1)	-3.796	766.65566	DGTS (36:1)	0.169
C47H84O7N	774.624779	774.62393	DGTS (37:4)	-0.388	774.62307	DGTS (37:4)	-1.498
C47H86O7N	776.640429	776.63693	DGTS (37:3)	-3.799	776.63989	DGTS (37:3)	0.012

Formula		Sample			Sample		
		Elysia viridis			*Elysia viridis* Starvation		
**Betaine lipids $[M+H]^+$**	***m/z* library**	***m/z* experiment**	**Lipid species (C:N)**	**Delta ppm**	***m/z* experiment**	**Lipid species (C:N)**	**Delta ppm**
C48H82O7N	784.609128	784.60859	DGTS (38:6)	0.012	784.60846	DGTS (38:6)	-0.153
C48H84O7N	786.624779	786.62356	DGTS (38:5)	-0.852	786.62364	DGTS (38:5)	-0.750
C48H86O7N	788.640429	788.63814	DGTS (38:4)	-2.207	788.63988	DGTS (38:4)	0.000
C50H80O7N	806.593479	806.59151	DGTS (40:9)	-1.761	806.59310	DGTS (40:9)	0.170
C50H82O7N	808.609128	808.60752	DGTS (40:8)	-1.311	808.60713	DGTS (40:8)	-1.794
